# Social Media Use and Body Image Disorders: Association between Frequency of Comparing One’s Own Physical Appearance to That of People Being Followed on Social Media and Body Dissatisfaction and Drive for Thinness

**DOI:** 10.3390/ijerph18062880

**Published:** 2021-03-11

**Authors:** Barbara Jiotsa, Benjamin Naccache, Mélanie Duval, Bruno Rocher, Marie Grall-Bronnec

**Affiliations:** 1 Addictology and Liaison Psychiatry Department, Nantes University Hospital, 44000 Nantes, France; barbara.jiotsa@hotmail.com (B.J.); naccache.benjamin@gmail.com (B.N.); bruno.rocher@chu-nantes.fr (B.R.); 2 Public Health Department, Nantes University Hospital, 44000 Nantes, France; melanie.duval@chu-nantes.fr; 3 Inserm UMR 1246, Nantes and Tours Universities, 44200 Nantes, France

**Keywords:** body image disorders, teenagers, social media, eating disorders, selfies, social comparisons, body dissatisfaction, drive for thinness

## Abstract

(1) Summary: Many studies have evaluated the association between traditional media exposure and the presence of body dissatisfaction and body image disorders. The last decade has borne witness to the rise of social media, predominantly used by teenagers and young adults. This study’s main objective was to investigate the association between how often one compares their physical appearance to that of the people they follow on social media, and one’s body dissatisfaction and drive for thinness. (2) Method: A sample composed of 1331 subjects aged 15 to 35 (mean age = 24.2), including 1138 subjects recruited from the general population and 193 patients suffering from eating disorders, completed an online questionnaire assessing social media use (followed accounts, selfies posted, image comparison frequency). This questionnaire incorporated two items originating from the Eating Disorder Inventory Scale (Body Dissatisfaction: EDI-BD and Drive for Thinness: EDI-DT). (3) Results: We found an association between the frequency of comparing one’s own physical appearance to that of people followed on social media and body dissatisfaction and drive for thinness. Interestingly, the level of education was a confounding factor in this relationship, while BMI was not. (4) Discussion: The widespread use of social media in teenagers and young adults could increase body dissatisfaction as well as their drive for thinness, therefore rendering them more vulnerable to eating disorders. We should consequently take this social evolution into account, including it in general population prevention programs and in patients’ specific treatment plans.

## 1. Introduction

Body image is defined as one’s perception, thoughts, and emotions revolving around one’s own body. It is the depiction of one’s body representation, including their mirror reflection, and it reflects social constructs, which depend on a society’s culture and norms. This conception is created using body ideals, substantially communicated via media, family, and peers.

For the last 30 years, media have been over-exposing people to thinness ideals, starting from a young age [1], turning this ideal into a new reference standard [2]. Young women, who are most sensitive to thinness ideals, tend to liken them to beauty and success [3]. Thus, etiologic models incorporating environmental factors consider social pressure about physical appearance to be a determining factor in developing eating disorders (EDs) [4,5].

However, even though this social pressure is indisputable, not all people are vulnerable to it. It is the degree with which they will relate to these thinness standards, namely how they internalize this ideal, that will help to predict the risk of developing an ED [6]. Indeed, internalizing thinness standards can lead to an alteration in body image, resulting in body dissatisfaction and exaggerated concerns about body and weight [4]. Body dissatisfaction is characterized by an inconsistency between one’s real body and the idealized body. It is one of the most studied psychological constructs in body image disorders literature [4,7,8,9]. According to the literature, it is often linked to psychological distress [10,11] and is a proven risk factor for developing an ED [12,13], through, in particular, the implementation of food restriction that can lead to anorexia nervosa (AN) [14,15] or to the onset of binge eating episodes (with or without compensatory behaviors to prevent weight gain). According to several authors, body dissatisfaction found in AN patients differs from that of control subjects by a greater feeling of inconsistency between their actual body and the desired body [16]. Indeed, in addition to overestimating the size of their actual shape, AN patients seek to resemble an ideal significantly thinner than subjects without EDs do. People with AN and bulimia nervosa share the same body image obsession, with the pervasive fear of gaining weight [4]. Finally, subjects with binge eating disorders tend to be overweight, or even obese, which can reinforce body dissatisfaction [17].

Social comparison, combined with the internalization of ideals, is one of the main mechanisms participating in one’s body image perception. These two mechanisms are instrumental in developing body dissatisfaction [1,18,19]. Several studies have shown that individuals who compare their physical appearance to that of others they considered to be more attractive than them, such as models or celebrities, had a higher chance of being dissatisfied with their body image and developing an ED [20,21,22,23].

Although historically speaking, body norms have been mainly conveyed through traditional media (TV, radio, newspaper, magazines), the last few years have borne witness to the rise and expansion of social media use. The term “social media” refers to every website and online mobile app with user-generated content. They enable their users to participate in online exchanges, broadcast self-made content, and join virtual communities. They are mostly used by teenagers and young adults, and the most common ones are Facebook, Instagram, Snapchat, and Twitter. Several studies have suggested that social media exposure could foster body dissatisfaction and result in risky eating behaviors by broadcasting thinness ideals individuals thus long for [18,24,25]. Among the identified mechanisms that explain this outcome, the most common ones are social comparison based on physical appearance and thinness ideals’ internalization through daily exposure to idealized bodies. Indeed, physical appearance holds a central place in social media today [26].

There is, to this day, a lack of scientific data, and in particular French data, about the association between the use of social media and risky eating behaviors [27]. In this context, this study’s main objective was to study the association between, on one hand, daily exposure to idealized bodies through social media and, on the other hand, the presence of two dimensions fostering body image disorders: body dissatisfaction and drive for thinness. A secondary objective was to compare two populations, one with a risk of suffering from ED, and the other one free of that risk, using different variables. The hypothesis was that at-risk participants were more dissatisfied with their physical appearance, had a higher drive for thinness, and compared themselves more often to social-media-conveyed images.

## 2. Materials and Methods

### 2.1. Study Design and Ethics Statements

This is a transversal observational study. Participants had to answer a questionnaire available online. Since it was an investigation involving the health field, but with an objective that did not involve the developing of biological or medical knowledge, it not fit in the French Jardé legal framework (and thus, approval from an ethics committee was not required). Data collection was made anonymously, was digitalized, and was realized outside of a care setting. Answering the questionnaire was interpreted as consent for data use, as it displayed that the results would be used in a survey, but that the participation would be anonymous, and that there were no data that would lead them to be recognized should they decide to participate.

### 2.2. Participants Recruitment

The study’s general population participants were enlisted via a social media publication (Facebook, Instagram, Twitter) and via posters in gyms. These posters were also sent to health workers with a practice in Nantes and in different French cities (psychiatrists, GPs, psychologists, etc.), who were tasked with informing their ED patients about this study. The Fédération Française Anorexie Boulimie (FFAB, French Federation for Anorexia and Bulimia), which is an association regrouping professionals working in the ED field, helped to broadcast the questionnaire using mailing lists, social media, and websites. Recruitment occurred between September 2019 and December 2019.

The inclusion criteria were as follows: using their Facebook and/or Instagram account daily and being 15 to 35 years old. This age range was chosen in light of the current literature, which shows that use of social media and body image concerns involved mainly teenagers and young people [28,29]. Moreover, participants recruited via a health professional had to register their ED diagnosis for which they were treated.

### 2.3. Evaluation

#### 2.3.1. General Data

The questionnaire’s first part was designed to register sex, age, degrees, and current height and weight to measure body mass index (BMI). 

#### 2.3.2. Social Media Use

The questionnaire’s second part interrogated the participants about their use of social media: platform, frequency (number of uses per day), time spent (hours per day), frequency of comparing one’s physical appearance to that of people followed on social media, and the frequency of posting “selfies” (a photograph that you take of yourself). 

#### 2.3.3. Body Image

The questionnaire’s third part evaluated body image perception, using the Eating Disorder Inventory-2 (EDI-2) scale, translated and adapted in French [30,31]. It is a self-rated questionnaire evaluating psychological characteristics and symptoms associated with ED, using 11 subscales. We used the “Drive for Thinness” subscale (EDI-DT), composed of 7 questions (score of 0 to 21), and “Body Dissatisfaction” subscale (EDI-BD), composed of 9 questions (score of 0 to 27). The subscales are presented in Table 1. 

#### 2.3.4. ED Screening

The questionnaire’s last part aimed at screening ED, using the Sick-Control-One Stone-Fat-Food (SCOFF) self-questionnaire. It is a simple survey of 5 questions used to screen eating disorders in general population [32]. The French validation depicted this questionnaire to be as efficient and relatable as the original, with a great sensitivity and specificity in diagnosing ED when a patient has a score of 2 or over [33]. It enabled us to sort the population sample into two groups depending on their risk of having an ED: when their score was ≥2, they were sorted in the “SCOFF positive” group, and when their score was <2, in the “SCOFF negative” group. The SCOFF questionnaire is presented in Table 2.

### 2.4. Statistical Analysis 

A descriptive statistical analysis was conducted for the entire sample. Continuous variables are described by means and standard deviations, while categorical variables are presented as numbers and percentages.

We asked all participants to complete the SCOFF questionnaire, so that they were sorted into two groups depending on their results: the “SCOFF+” group gathering all participants with a SCOFF score of 2 or over, and therefore with the risk of suffering from an ED, and the “SCOFF−” group gathering all participants with a SCOFF score under 2. These two groups were then compared based on all collected variables. We applied a Student’s *t*-test for quantitative variables (“age”, “EDI-BD”, “EDI-DT”, and “average BMI”), a Chi-squared test for qualitative variables (“sex”, “level of education”, “social media use frequency”, “time spent”, “body comparison”, “groups of BMI”), and Fisher exact test for multimodal qualitative variables whose theoretical headcount did not allow the use of the Chi-squared test (“posting selfies”).

Then, we looked for an association between the frequency of comparing one’s own physical appearance to that of people followed on social media and the scores measured using the EDI Body Dissatisfaction and Drive for Thinness subscales. We thus performed two linear regressions with adjustment for two potential confounding factors (BMI and level of education). Confounding factor status was assessed by searching for an association of the two variables with EDI subscores on the one hand and with the frequency of comparing one’s own physical appearance to that of people followed on social media on the other hand.

The significance threshold for all these analyses was set at *p* = 0.05 (α risk of 5%).

Statistical analyses were done using the SPSS software (Statistical Package for Social Science, IBM, Armonk, NY, USA).

## 3. Results

### 3.1. Population Description

**Figure 1:**
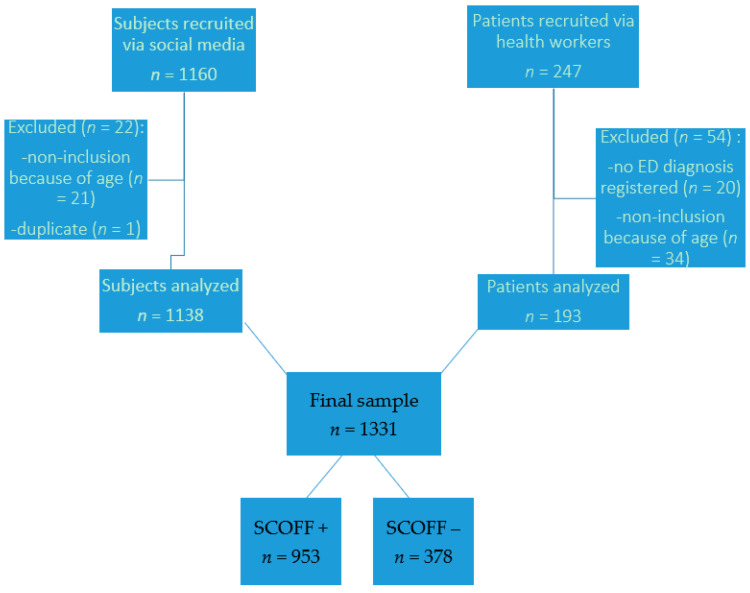
Flow chart of subjects’ inclusion.

In total, 1407 questionnaires were completed, and 1331 were analyzed. A total of 1138 subjects were from the general population, and 193 were ED patients recruited via health workers. Seventy-six completed questionnaires (5.4%) were excluded from the analysis because they did not match the age criteria or because their ED diagnosis was not communicated (for ED patients recruited via health workers). Figure 1 represents the study’s flowchart. 

The participants’ age ranged from 15 to 35 (M = 24.2, σ = 4.2). Most were women (97.7%). They had, for the most part, a Bachelor’s degree. Mean BMI was 22.3 (σ = 4.2).

Table 3 presents the final sample’s characteristics.

Most participants declared using Facebook (93%) and Instagram (92.8%). Other social media were less frequently used: Snapchat (68.4%), Twitter (29.1%), and Tiktok (2.5%).

In total, 57.3% of participants had a private account and 42.7% an account open to the public. Users declared that they used social media mainly to “like posts” (82.7%) and to “observe content, as *ghost followers* (bots or inactive accounts)” (65.4%). In total, 92.7% said that they used social media to “follow friends and acquaintances”, “follow healthy food content” (68%), “follow the news” (67%), and “follow fitness content” (61.2%). 

Regarding participants recruited via health workers for whom data were analyzed (N = 193), the most frequently reported ED was anorexia nervosa restricting type (41%), followed by anorexia nervosa purging type (28%), binge eating disorder (16%), bulimia nervosa (12%), and unspecified feeding or eating disorder (9%). 

### 3.2. Comparing Participants Based on Their ED Screening

The final sample was sorted into two groups according to the SCOFF’s results (*n* = 953 in the SCOFF+ group; *n* = 378 in the SCOFF− group). These groups were compared using all described variables, and the results are showcased in Table 3. 

SCOFF+ group subjects had a significantly higher social media use (regarding both frequency and time spent), a significantly higher frequency of comparing their physical appearance to that of people they followed, and of posting selfies. 

In addition, they declared having significantly higher EDI-BD and EDI-DT scores than SCOFF− subjects (*p* < 0.001), and they more frequently had BMI both in the lower and higher ranges. 

### 3.3. Association between the Frequency of Comparing One’s Own Physical Appearance to That of People Followed on Social Media and EDI Body Dissatisfaction and Drive for Thinness

In the search for confounding factors associated with both the frequency of comparing one’s own physical appearance to that of people followed on social media and EDI-BD and EDI-DT scores, we found a significant association between the level of education and the frequency of comparing one’s own physical appearance to that of people followed on social media (Table 4). Similarly, we observed an association between the modality “Level of education ≥12” and EDI-BD: participants with a level of education ≥12 had a mean EDI-BD score 2.5 points lower compared to that of participants with a level of education <12 (Table 5). We also found a similar association between the modality “Level of education ≥12” and EDI-DT: participants with a level of education ≥12 had a mean EDI-DT score 3 points lower compared to that of participants with a level of education <12 (Table 6).

Furthermore, we did not find any significant association between BMI and the frequency of comparing one’s own physical appearance to that of people followed on social media (Table 7). A significant but very weak correlation (<0.3) was found between the BMI and the two EDI subscores (Table 8). In view of these results, we did not retain BMI as a confounding factor for the following analysis.

The results of the search for an association between the frequency of comparing one’s own physical appearance to that of people followed on social media and EDI Body Dissatisfaction and Drive for Thinness scores are presented in Table 9 and Table 10. As showcased in Table 9, the “Sometimes”, “Often”, and “Always” frequency of comparing modalities were significantly associated with the EDI-DT score. Participants who sometimes compared their own physical appearance to that of people followed on social media had a mean EDI-DT score 2.0 points higher than that of those who never compared themselves; those who often compared themselves had a mean EDI-DT score 5.3 points higher; and those who always compared themselves had a mean EDI-DT score 8.4 points higher.

In addition, according to Table 10, the “Often” and “Always” frequency of comparing modalities were significantly associated with the EDI-BD score. Participants who often compared their own physical appearance to that of people followed on social media had a mean EDI-BD score 5.6 points higher than that of those who did not, and those who always compared themselves to social media images had an average EDI-BD score 9.2 points higher than that of those who never did.

## 4. Discussion

### 4.1. Discussing the Main Results

Our survey aimed to study the links between social media use, body image disorders, and ED prevalence in a teenage and young adult population.

First, we found that ED or at-risk of ED subjects presented significantly different results concerning all social media use parameters. Using platforms such as Facebook and Instagram has been particularly associated with a higher body dissatisfaction and the appearance of ED symptoms [27,34]. As was expected, in ED or at-risk of ED patients, Body Dissatisfaction rates were higher, as was their Drive for Thinness. A common ED assumption is that ED patients develop a cognitive structure that focalizes on weight, combined with, most of the time, a mistaken perception of their own body image, especially in anorexia nervosa. These subjects tend to yearn for a thinner body ideal than the general population, thus creating a substantial inconsistency between what they think they look like and what they yearn to look like [35]. Leahey and her colleagues in 2011 [36] found that, in addition to increasing body dissatisfaction, social comparisons have an influence on negative effects, guilt, as well as diets and physical-activity-centered thoughts.

Participants in general were seldom prone to posting selfies. Ridgway and her colleagues [37] conducted in 2018 a study on Instagram and posting selfies, which showed that a higher body image satisfaction was associated with an increase in posting selfies. This could explain the low percentage of self-promoting subjects found in this study.

Second, we confirmed the existence of a significant association between, on one hand, the frequency of comparing one’s own physical appearance to that of people followed on social media and, on the other hand, Body Dissatisfaction and Drive for Thinness scores measured using the EDI scale. It seems that the more the subjects compared themselves to the images, the more they increased their body dissatisfaction and their drive for thinness. However, this association can work two ways. Indeed, it could be that the depth of body dissatisfaction and the drive for thinness increase the inclination to compare oneself to images. Our results are in accordance with those found in the literature, which identified a link between social media use and body image disorders [26,38,39]. It has also been found that subjects who often compared their physical appearance to that of idealized images were more dissatisfied with their body and had a higher drive for thinness than those who compared themselves less often [40,41]. Interestingly, the level of education was a confounding factor in this relationship, while BMI was not. Indeed, the relation between frequency of comparing one’s own physical appearance to that of people followed on social media on the one hand and EDI DT and BD subscores on the other hand is modified by the level of education, starting from a level corresponding to a Bachelor’s degree (>12 + 3 years).

Self-assessment is a fundamental reflexive analysis tool [42]. It plays an essential part in self-positioning among others and oneself. This self-evaluation must resort to social comparisons, which have a direct link to self-esteem. Body image’s sociocultural construct takes shape using body ideals that are broadcasted through, in particular, media, family, and peers and are thereafter internalized by individuals [43]. Reaching these body norms is usually perceived as proof of self-control and success, which leads one to stand out from the crowd in a positive way [44]. Internalizing body ideals thus creates an authentic concern for one’s physical appearance, which will be observed and judged by others [45]. This can trigger body dissatisfaction, which usually involves feeling inadequate in one’s body, estranged from the ideal one pursues [43]. Fear of gaining weight can be exacerbated when thinness is one of narcissism’s only tools. It can lead to behaviors such as food restriction, excessive physical activity, with the aim of modifying one’s appearance and thus fit into social standards. This excessive self-surveillance can bring about emotional and psychological consequences, including shame about one’s own body, self-bashing, anxiety, and depression, up to ED [46].

Finally, although estimating ED prevalence in a young adult population was not an objective determined beforehand, we must point out that most participants had a SCOFF+ result (71%), suggesting they might suffer from an ED. This questions whether a more systematic ED screening should be done in teenage and young adult populations, which are ED’s main targets. Several studies in which teenagers were interviewed have shown that they often are dissatisfied with their bodies, feeling like they are “too fat”, and most of them have already followed a diet [47,48,49]. These diets can include ingesting smaller portions, eating healthier food, up to major food restrictions and complete removal of some types of food, which can be found in ED.

### 4.2. Study’s Strengths and Weaknesses

There are several limits to this study. First, it is a transversal study, which cannot prove the existence of a causal relationship between the studied variables. Therefore, longitudinal studies are necessary in finding out how this association works. Second, the online questionnaire was not designed to collect data that could be considered as indicators of individual or family vulnerabilities for ED, which did not allow for stratified analyses. Third, measuring the time spent on social media and how often participants used it was done through self-reported data, which could induce a declaration bias, thus limiting the data’s precision. Future studies could use technologies such as data tracking (virtual counter measuring connection frequency and time spent) in order to have more precise data and thus be more confident in the data’s reliability. Fourth, the participants’ recruitment induced a selection bias. Indeed, having used daily use of social media as an inclusion criterion leads to selecting a certain type of population and renders irrelevant any extrapolation to the general population. Moreover, recruiting via gyms may have led to selecting individuals with a specific concern for their body image. We can assume that these subjects, who paid specific attention to their physical appearance, might have certain demands concerning themselves, which might involve body dissatisfaction and an exaggerated drive for thinness. The daily use of social networks could also be a reflection of excessive body concerns, which could lead to more body dissatisfaction and a more pronounced drive for thinness compared to subjects who are less exposed to these kinds of media. Fifth, our participants recruited via health workers may not be representative of all ED patients for several reasons: ED diagnosis was self-reported, anorexia nervosa restricting type was overrepresented in our sample, and the most severe patients may not be psychologically available to participate in a study like this one. Finally, the SCOFF questionnaire is a screening tool and not a diagnostic one. It does not enable discriminating between anorexia nervosa, bulimia nervosa, or binge eating disorder among participants, but we can assume that all types of ED were present in the SCOFF+ group, as the participants in this group more frequently had BMI both in the lower and higher ranges.

However, these limits are balanced by the study’s strengths. First, the sample rallied a significant number of participants, and their sorting into two groups after ED screening was quite proportionate, which ensured the statistical analyses’ power. Second, EDs were screened using a validated tool for the general population, and the Body Dissatisfaction and Drive for Thinness dimensions were evaluated using a self-questionnaire whose psychometric characteristics have been validated in clinical populations. Finally, to the extent of our knowledge, this type of study had never been conducted in France, thus bringing forth unprecedented data.

### 4.3. Perspectives

This study’s results open new avenues for clinicians to explore social media use and cognitive pathways in ED. Indeed, social media exposure and, in particular, exposure to edited and idealized images could contribute to inaccurate thought processes about body image, internalizing what is socially valued on social media as a personal goal. Since we know that cognitive pathways play an important part in ED development and continuation [50], it seems relevant to explore patients’ use of social media and the cognitions associated. This could contribute to increasing psychotherapy’s efficacy, enriching prevention programs using cognitive dissonance, therapies that have been proven to be effective in reducing ED symptoms’ intensity [51]. A way to implement this could be to encourage the development of the ability to question social media, encouraging patients to think of arguments that go against posting idealized photos on social media [27].

When considering the general population, when we see how important social comparison based on physical appearance is in developing body dissatisfaction, prevention programs could be useful. It seems relevant to encourage teenagers, particularly those with the tendency to compare themselves to their peers, to evaluate their body using health criteria instead of using other peoples’ bodies as a standard. Additionally, it would be interesting to intervene by deconstructing the “ideal body” myth, with the goal of diminishing the comparison to “idols”. Finally, it seems relevant to inform people that some role models’ BMI and body type are not representative of those of most of the population and that trying to reach their body type could be harmful. ED screening in this population should thus be more systematic.

## 5. Conclusions

To summarize, we found an association between the frequency of comparing one’s own physical appearance to that of people followed on social media and body dissatisfaction and drive for thinness. Interestingly, the level of education was a confounding factor in this relationship, while BMI was not. The widespread use of social media in teenagers and young adults could increase body dissatisfaction as well as their drive for thinness, therefore rendering them more vulnerable to eating disorders.

## Figures and Tables

**Table 1:** Drive for Thinness and Body Dissatisfaction subscales of Eating Disorder Inventory-2.

Drive for Thinness	Always (=3)	Usually (=2)	Often (=1)	Sometimes (=0)	Seldom (=0)	Never (=0)
1—I eat sweets and carbohydrates without feeling nervous	*□*	*□*	*□*	*□*	*□*	*□*
2—I think about dieting	*□*	*□*	*□*	*□*	*□*	*□*
3—I feel extremely guilty after overeating	*□*	*□*	*□*	*□*	*□*	*□*
4—I am terrified of gaining weight	*□*	*□*	*□*	*□*	*□*	*□*
5—I exaggerate or magnify the importance of weight	*□*	*□*	*□*	*□*	*□*	*□*
6—I am preoccupied with the desire to be thinner	*□*	*□*	*□*	*□*	*□*	*□*
7—If I gain a pound, I worry that I will keep gaining	*□*	*□*	*□*	*□*	*□*	*□*
**Body Dissatisfaction**	**Always**	**Usually**	**Often**	**Sometimes**	**Seldom**	**Never**
1—I think that my stomach is too big (+)	*□*	*□*	*□*	*□*	*□*	*□*
2—I think that my thighs are too large (+)	*□*	*□*	*□*	*□*	*□*	*□*
3—I think that my stomach is just the right size (−)	*□*	*□*	*□*	*□*	*□*	*□*
4—I feel satisfied with the shape of my body (−)	*□*	*□*	*□*	*□*	*□*	*□*
5—I like the shape of my buttocks (−)	*□*	*□*	*□*	*□*	*□*	*□*
6—I think my hips are too big (+)	*□*	*□*	*□*	*□*	*□*	*□*
7—I think that my thighs are just the right size (−)	*□*	*□*	*□*	*□*	*□*	*□*
8—I think my buttocks are too large (+)	*□*	*□*	*□*	*□*	*□*	*□*
9—I think that my hips are just the right size (−)	*□*	*□*	*□*	*□*	*□*	*□*

**Table 2:** Sick-Control-One Stone-Fat-Food (SCOFF) questionnaire.

	Yes	No
1—Do you make yourself sick because you feel uncomfortably full?	□	□
2—Do you worry you have lost control over how much you eat?	□	□
3—Have you recently lost over 1 stone (14 lb) in a 3-month period?	□	□
4—Do you believe yourself to be fat when others say you are too thin?	□	□
5—Would you say that food dominates your life?	□	□

Yes = 1 point; score of ≥2 suggests an eating disorder.

**Table 3:** Final sample characteristics and comparison between SCOFF+ and SCOFF− groups.

	Final Sample (*n* = 1331)		SCOFF− (*n* = 378)		SCOFF+ (*n* = 953)		*p* Value
	Mean or Number of Participants	Standard Deviation or Percentage	Mean or Number of Participants	Standard Deviation or Percentage	Mean or Number of Participants	Standard Deviation or Percentage	
**SOCIODEMOGRAPHIC CHARACTERISTICS**							
**Age**	24.2	4.2	25.1	4.2	23.9	4.2	<0.001 ***
							(Student’s *t*-test)
**Sex**							0.012 *
Female	1300	97.7%	363	96.0%	937	98.3%	(Chi-squared test)
Male	31	2.3%	15	4.0%	16	1.7%	
**Studies level**							<0.001 ***
Less than Level 12	71	5.3%	16	4%	55	6%	(Chi-squared test)
Level 12	229	17.2%	62	16%	167	18%	
Level 12 + 2 years	208	15.6%	50	13%	158	17%	
Level 12 + 3 (Bachelor’s degree)	320	24.0%	89	24%	231	24%	
Level 12 + 5 (Master’s degree)	380	0.285	96	25%	284	30%	
Degree over Level 12 + 5	123	0.092	65	17%	58	6%	
**SOCIAL MEDIA USE**							
**Frequency**							<0.001 ***
Max. once a day	64	5%	17	4%	47	5%	(Chi-squared test)
2 to 10 times a day	578	43%	194	51%	384	40%	
10 to 20 times a day	439	33%	115	30%	324	34%	
Over 20 times a day	250	19%	52	14%	198	21%	
**Time spent**							0.010 **
Less than 1 h	232	17%	81	21%	151	16%	(Chi-squared test)
Between 1 and 5 h	1048	79%	289	76%	759	80%	
Over 5 h	51	4%	8	2%	43	5%	
**Body comparison**							<0.001 ***
Never	33	2%	18	5%	15	2%	(Chi-squared test)
Seldom	114	9%	56	15%	58	6%	
Sometimes	317	24%	130	34%	187	20%	
Often	523	39%	133	35%	390	41%	
Always	344	26%	41	11%	303	32%	
**Posting selfies**							<0.001 ***
Never	457	34%	146	39%	311	33%	(Fisher exact test)
1 or 2 times a month	756	57%	199	53%	557	58%	
Once a week	93	7%	24	6%	69	7%	
3 to 4 times a week	18	1%	7	2%	11	1%	
Daily	7	1%	2	1%	5	1%	
**EATING DISORDERS**							
**EDI-BD**	12.4	7.5	7.9	6.6	14.2	7	<0.001 ***
							(Student test)
**EDI-DT**	8.9	6	4.1	4.2	10.8	5.5	<0.001 ***
							(Student test)
**Average BMI**	22.3	4.2	22.2	3.5	22.3	4.5	0.575
							(Student test)
**Categories of BMI**							<0.001 ***
<17.5	96	7.2%	9	2.4%	87	9.1%	(Chi-squared test)
[17.5–25]	981	73.7%	306	81.0%	675	70.8%	
≥25	254	19.1%	63	16.7%	191	20.0%	

Note. BDI: body mass index; EDI-IC: Eating Disorder Inventory—Body Dissatisfaction; EDI-RM: Eating Disorder Inventory—Drive for Thinness. *: *p* < 0.05; **: *p* < 0.01; ***: *p* < 0.001. According to the International Classification of Diseases, anorexia nervosa is associated with a BMI < 17.5.

**Table 4:** Association between level of education and frequency of comparing one’s own physical appearance to that of people followed on social media.

	Chi-Squared Test	*p*-Value
Frequency of comparing one’s own physical appearance	38.165	0.008 **

Note. **: *p* < 0.01.

**Table 5:** One-way ANOVA results looking for a link between EDI-BD score and level of education.

	Estimates	*p*-Value
Intercept	13.620	<2 × 10^−16^ ***
Studies level: Less than level 12		
Studies level: Level 12	−0.672	0.507
Studies level: Level 12 + 2 years	−0.778	0.447
Studies level: Level 12 + 3 (Bachelor’s degree)	−1.560	0.110
Studies level: Level 12 + 5 (Master’s degree)	−1.307	0.175
Degree over Level 12 + 5	−2.538	0.022 *

Global *p*-value = 0.1338. Note: The modality “Less than level 12” was chosen as the reference modality for this analysis. *: *p* < 0.05; ***: *p* < 0.001.

**Table 6:** One-way ANOVA results looking for a link between EDI-DT score and level of education.

	Estimates	*p*-Value
Intercept	10.141	<2 × 10^−16^ ***
Studies level: Less than level 12		
Studies level: Level 12	−0.730	0.368
Studies level: Level 12 + 2 years	−0.477	0.561
Studies level: Level 12 + 3 (Bachelor’s degree)	−1.328	0.090
Studies level: Level 12 + 5 (Master’s degree)	−1.451	0.061
Degree over Level 12 + 5	−3.019	0.0007 ***

Global *p*-value = 0.0016. Note: The modality “Less than level 12” was chosen as the reference modality for this analysis. ***: *p* < 0.001.

**Table 7:** One-way ANOVA results looking for a link between BMI and frequency of comparing one’s own physical appearance to that of people followed on social media.

	Estimates	*p*-Value
Intercept	21.109	<2 × 10^−16^ ***
Body comparison: Never		
Body comparison: Seldom	1.002	0.233
Body comparison: Sometimes	1.049	0.177
Body comparison: Often	1.155	0.130
Body comparison: Always	1.384	0.074

Global *p*-value = 0.4368. Note: The modality “Never” was chosen as the reference modality for this analysis. ***: *p* < 0.001.

**Table 8:** Results of association between BMI and EDI scores.

	Coefficient de Correlation de Pearson Avec son IC à 95%	*p*-Value
EDI-DT	0.071 [0.017; 0.1239]	0.0099 **
EDI-BD	0.253 [0.202; 0.302]	<0.001 ***

Note. EDI-BD: Eating Disorder Inventory—Body Dissatisfaction. **: *p* < 0.01; ***: *p* < 0.001.

**Table 9:** Linear regression looking for a link between EDI-DT score and frequency of comparing one’s own physical appearance to that of people followed on social media.

	Estimates	*p*-Value
Intercept	5.859	8.7 × 10^−8^ ***
Body comparison: Never		
Body comparison: Seldom	0.438	0.678
Body comparison: Sometimes	2.021	0.038 *
Body comparison: Often	5.314	3.4 × 10^−8^ ***
Body comparison: Always	8.421	<2.2 × 10^−16^ ***
Studies level: Less than level 12		
Studies level: Level 12	−1.399	0.053
Studies level: Level 12 + 2 years	−1.415	0.0539
Studies level: Level 12 + 3 (Bachelor’s degree)	−1.723	0.0138 *
Studies level: Level 12 + 5 (Master’s degree)	−1.999	0.0038 **
Degree over Level 12 + 5	−2.936	0.0002 ***

Global *p*-value <2.2 × 10^−16^ ***. Note: Modalities “Less than level 12” and “Never” were chosen as the reference modalities for this analysis. *: *p* < 0.05; **: *p* < 0.01; ***: *p* < 0.001.

**Table 10:** Linear regression looking for a link between EDI-BD score and frequency of comparing one’s own physical appearance to that of people followed on social media.

	Estimates	*p*-Value
Intercept	9.087	1.1 × 10^−10^ ***
Body comparison: Never		
Body comparison: Seldom	1.225	0.365
Body comparison: Sometimes	1.768	0.158
Body comparison: Often	5.564	6.5 × 10^−6^ ***
Body comparison: Always	9.226	2.4 × 10^−13^ ***
Studies level: Less than level 12		
Studies level: Level 12	−1.437	0.122
Studies level: Level 12 + 2 years	−1.785	0.058
Studies level: Level 12 + 3 (Bachelor’s degree)	−1.986	0.027 *
Studies level: Level 12 + 5 (Master’s degree)	−1.940	0.029 *
Degree over Level 12 + 5	−2.471	0.016 *

Global *p*-value <2.2 × 10^−16^ ***. Note: Modalities “Less than level 12” and “Never” were chosen as the reference modalities for this analysis. *: *p* < 0.05; ***: *p* < 0.001.

## Data Availability

The data presented in this study are available on request from the corresponding author.
